# Human Acellular Dermal Matrix in Reconstructive Surgery—A Review

**DOI:** 10.3390/biomedicines10112870

**Published:** 2022-11-09

**Authors:** Marcin Gierek, Wojciech Łabuś, Diana Kitala, Andrzej Lorek, Gabriela Ochała-Gierek, Karolina Mikuś Zagórska, Dariusz Waniczek, Karol Szyluk, Paweł Niemiec

**Affiliations:** 1 Dr Stanislaw Sakiel Burn Treatment Centre in Siemianowice Slaskie, 41-100 Siemianowice Slaskie, Poland; 2 Department of Surgical Oncology, University Medical Center, Silesian Medical University, ul. Ceglana 35, 40-514 Katowice, Poland; 3 Dermatology Department, City Hospital in Sosnowiec, ul. Zegadłowicza 3, 41-200 Sosnowiec, Poland; 4 Department of Physiotherapy, Faculty of Health Sciences in Katowice, Medical University of Silesia in Katowice, 40-752 Katowice, Poland; 5 Department of Orthopaedic and Trauma Surgery, District Hospital of Orthopaedics and Trauma Surgery, 41-940 Piekary Śląskie, Poland; 6 Department of Biochemistry and Medical Genetics, Faculty of Health Sciences in Katowice, Medical University of Silesia in Katowice, 40-752 Katowice, Poland

**Keywords:** acellular dermal matrix, bioengineering, allograft

## Abstract

Reconstructive surgery often confronts large tissue defects. This creates a need to look for materials that are immunogenic but offer the possibility of tissue filling. ADM—acellular dermal matrix—is a biological collagen matrix without immunogenicity, which is more commonly used in surgical treatment. Reconstructive surgery is still searching for various biocompatible materials that can be widely used in surgery. The available materials have their advantages and disadvantages. This paper is a literature review on the use of human acellular dermal matrix (ADM) in reconstructive surgery (surgical oncology, plastic and reconstructive surgery, and gynecologic reconstructive surgery). ADM appears to be a material of increasing use in various fields of surgery, and thus, further research in this area is required.

## 1. Introduction

The adaptation of the human body in response to injury and damage enhances our skill in tailoring procedures that optimize long-term treatment outcomes, aesthetic aspects of wound formation, and the incidence of disease. An understanding of these processes is crucial in reconstructive surgery.

Large skin defects often require reconstructive methods to close the wound, and there is still a lack of biological materials that can be applied easily in such cases. A great advance in the field of plastic and other reconstructive surgery has been the introduction and successful use of acellular dermal matrices (ADMs) [1]. There is a great need for biological dressings for wound coverage in large skin defects, e.g., in the treatment of burns. Many of them include amniotic membranes, which are used widely during surgical treatment [2].

There are three groups of ADM [3]. These groups of biological matrices are obtained from skin donation (allografts), are obtained from animals (xenografts), or are obtained from plants. ADMs of synthetic origin are produced by technological processes like polymerization, composite ADMs are a biological-synthetic hybrid. [3]. Commercial ADM products are classified as a Class III medical device by European Union regulatory law and require the CE mark (the CE mark indicates that the product can be traded freely in any part of the European Economic Area). hADM (Human Acellular Dermal Matrix) is not significantly changed in its structure when obtained from human skin, which means that this material is classified as banked human tissue. In that case, it does not require approval by the FDA (Food and Drug Administration) in the USA [4].

The process of removing cells from tissues and organs is an important issue that has been described extensively in the literature [1,2,3,4,5].

The removal of cells from allografts results in an acellular collagenous mesh without immunogenicity that can be de novo revitalized by autologous cells. This kind of biological matrix (ADM) can activate regeneration mechanisms [1,2,3,4,5,6,7,8,9,10,11].

The immune system’s response is targeted against cell membrane lipids and proteins. Harvesting cells from tissue is an encouraging method to prevent the occurrence of graft vs host disorders, in which immune cells recognize the host as foreign in the recipient body’s cells. [12,13,14,15]. The process of decellularization, which involves removing cellular components, should minimize immune-induced inflammation, which can impair the biodegradation process of the transplanted biomaterial [13]. The process of removing cells from allogeneic human dermis creates a cell-free matrix that is not immunogenic. It is made of extracellular matrix (ECM) structures that can be repopulated with the patient’s own cells. This decellularized dermal matrix (ADM) can introduce normal proregenerative mechanisms in the human body [9,10,11,12,13,14,15,16].

Many studies have shown that cell-free extracellular matrix scaffolds are capable of proliferating and promoting the growth and differentiation of many kinds of cell populations in vitro, as well as inducing structural tissue remodeling processes after transplantation in humans [4,5,6,7,8].

Therefore, the use of 3-D ECM meshes derived from tissues is an increasingly used method in reconstructive surgery, regenerative medicine, and bioengineering [1,3,9,10,11,12,13,14,15,16,17].

Additionally, it can be assumed that ADMs have some advantages over “traditional” forms of therapy. In this approach, it should be remembered that autologous, native transplants are the optimal method of treatment, but the frequent lack of an adequate amount of healthy tissue or the wound created after autologous graft retrieval may be considered a serious limitation of this technique. As a result, artificial materials might be considered an optimal alternative. However, it is important to note a warning from the Food and Drug Administration (FDA) accordingly, as many authors have concluded serious complications (e.g., erosion of the mesh, recurrence) as a result of artificial material use [18,19,20]. In order to avoid the unfavorable side effects linked with the use of artificial materials, the use of biomaterials is more often chosen in general regenerative medicine. In these aspects, cell-free tissue grafts are a very interesting option. We are still looking for ideal materials that will be biocompatible. These criteria are met by the acellular dermal matrix. [5,6,7,8,9].

**Figure 1:**
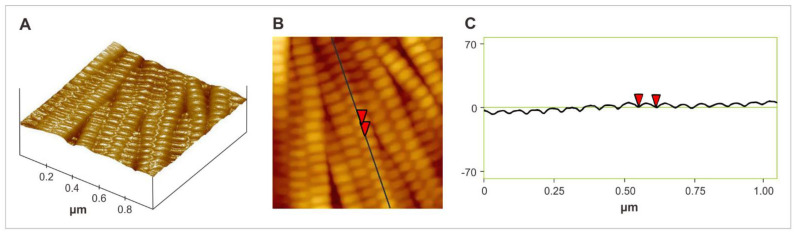
ADM image obtained with atomic force microscopy (native material): (**A**) 3D image of ADM; (**B**) “Height” image of ADM; (**C**) topography of the collagen fiber of the ADM according to the profile marked in Figure 1B (native material).

In Figure 1, an image of atomic force microscopy is shown, and is also presented in a 3D collagen structure image.

Acellular dermal matrices can be effectively used in several applications, and they must allow host blood vessels to grow in via angiogenesis into the voids left by ADM [1]. Soon after vascularization, ADMs integrate with the human body and become living connective tissue. The first mention of ADM usage was for skin replenishment in burn patients. Shortly after, the use of ADMs expanded to other diverse and various clinical uses. As a result of the unique characteristics of biological implants and the variety of available products, ADM can be very helpful in a range of applications within the field of plastic and reconstructive surgery or surgical oncology. There are many reports of novel applications of ADM in surgery. However, this review will focus on the areas that are best studied in the literature.

This manuscript is a review of the clinical applications of human ADMs in reconstructive surgical specialties. On the market, there are many commercial ADM products; however, some of the authors used their own in-house ADMs banked in tissue banks (e.g., Center for Burns Treatment in Siemianowice Śląskie, Poland). In Table 1, we attached the most popular commercial ADM products with a brief description of their characteristics.

## 2. Decellularization of Human Allogeneic Skin

**Figure 2:**
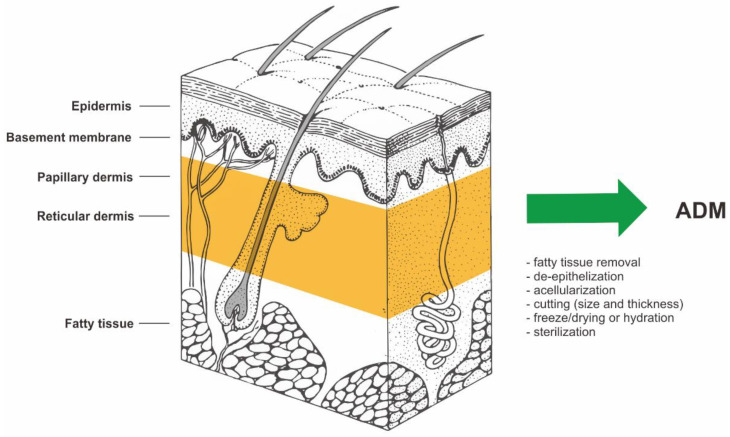
The biological source and the manufacturing process of ADM (acellular dermal matrix). Structure of human skin based on https://visualsonline.cancer.gov/details.cfm?imageid=2496 (access on 1 January 2001) [10].

ADM is made by taking a full-thickness section of skin from a donor source. In most cases, this is a human cadaver. However, porcine or bovine dermis is used as well. The process of cell removal from tissues and organs has been described extensively and in detail in previous literature [1,2,3,4,5,6,7,8,9]. The material for ADM preparation is allogeneic human skin procured during multi-organ/multi-tissue donations from the deceased donor. In further stages of preparation, allogeneic human skin is subjected to the action of selected cell-removing factors (e.g., proteolytic enzymes, detergents, etc.). Removal of cells from allogeneic human dermis yields an acellular mesh made of collagen which is non-immunogenic. [1,2,3,4,5,6,7,8,9]. The biological source and the manufacturing process of ADM is shown in Figure 2.

## 3. Burns and Wound Management

The organ with the largest surface area in the human body is the skin. That makes the skin very important. Reconstruction of skin continuity after a very severe burn is crucial for healing. Reconstruction after a severe burn injury also involves healing of the skin’s natural elasticity, texture, and contour. Complete destruction of the skin requires the use of a skin substitute. The method of choice is a graft consisting of the epidermis and a superthin layer of dermis. It is important to rebuild and replace this tissue to achieve good epithelial coverage [1,2,3,4,5,6,7,8].

The optimal method of treating extensive full-thickness (III) skin burns is split-thickness skin grafts (STSGs). STSGs are collected from an undamaged site for wound closure [1,2,3,4,5,6,7,8]. The main limitation of this method is the lack of healthy, undamaged skin that could be used as a graft. Moreover, the donor fields constitute an additional wound that weighs down the organism [9]. The sites from which the skin was removed for transplantation cause pain, therefore requiring the use of analgesic therapy, and leave scars while healing [6,9]. The limitations related to the use of autologous slow split-thickness skin grafts necessitate the search for new methods of treating severely burned patients. An alternative clinical standard that has been available for over three decades in the treatment of extensive full-thickness burns of the skin in patients with a deficit of donor sites is the use of in vitro cultured autologous keratinocytes (CEA) [5,6]. The effect of this method is the acceleration of the coating regeneration process, while long-term follow-up may develop functionally unacceptable and cosmetically hypertrophic scars [5,6,10,12]. Another alternative treatment for burns is the use of allogeneic skin grafts (allografts). This method has been used for over 70 years. Clinical indications for allogeneic skin grafts include protection against the drying out of the wound. Another indication is protection against infection and a mechanical barrier that prevents the loss of heat, proteins, and electrolytes. Although the use of allogeneic skin grafts is a temporary solution, it remains widely used in the treatment of burns. This kind of transplant is a temporary biological dressing. Almost always, this allograft is rejected by the human body (the recipient’s) after some time [7,8,13].

In the beginning, acellular dermal grafts were invented for use in the treatment of full-thickness burns (third-degree burns). The management of badly burned patients is complicated when donor skin is not available. When ADM is applied to a full-thickness skin defect with an overlying very thin split skin graft, migration and ingrowth of cells into the ADM are observed from both the skin graft and the underlying tissue and peripheral skin margins [14]. It all provides laminin for adhesion and type IV collagen, which is needed for epithelialization. The processing of allogeneic grafts results in scaffolding, which preserves normal collagen structure. Acellular vascular channels are preserved in this way as well [15]. In optimal conditions, vascular channels are repopulated by the host within a week, and the endothelium is being rebuilt [15]. ADMs eliminate the need for graft harvesting, which reduces scarring at the donor site that is seen with skin grafts [16].

**Figure 3:**
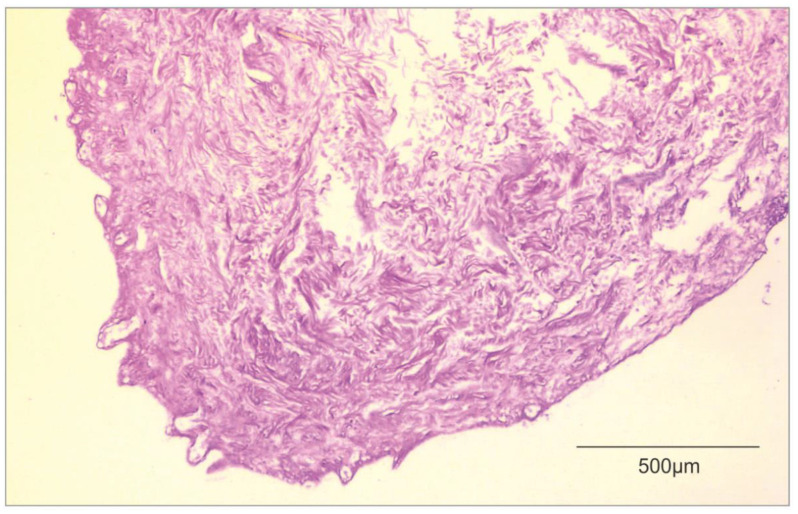
Histological image of ADM (own material).

In Figure 3, we attached a histological image of ADM.

This kind of biological dressing has become more popular in burn treatment indications over the past nearly 30 years. This skin substitute is used for open soft tissue defects like burns. Skin grafting is probably a less expensive technique but is often associated with very painful donor site. Application of ADM can undoubtedly improve patients’ quality of life after surgery. AlloDerm has been reported in the literature as a dermal substitute in full-thickness burn wounds [16,18].

## 4. Reconstructive Breast Procedures

Breast cancer is the most common form of cancer in the population and is the second most common cause of death in women. There are many literature reports on the use of ADM in breast reconstructive surgery [19]. Reconstructive breast surgery after a mastectomy is very limited. Women are more likely to choose reconstructions with implants rather than reconstructions based on autologous tissue transfer. This is due to the occurrence of side effects (i.e. a very painful donor site) [20]. Acellular dermal matrices are well-known biological scaffolds of human, bovine, or porcine origin that are increasingly used in the fields of plastic surgery and surgical oncology, mainly in breast reconstruction after conservative mastectomy in order to support implants [21].

In 2001, Duncan et al. reported the first use of ADM in breast surgery. Duncan et al. showed that visible implant undulation was decreased in a revision of primary augmentation with capsulectomy and ADM application [22]. The first published report mentioning the use of ADM in breast reconstruction came in 2005 (Breuing et al.). Breuing documented ten cases of patients who had undergone mastectomies with single-stage reconstruction and AlloDerm application [23]. This led to an increase in the use of ADM in breast reconstruction, making it one of the most popular applications. Reconstruction with tissue expanders and implants is a multi-step process that can be very difficult for patients. A main limitation of immediate single-stage reconstruction has always been the inability to completely cover the implant with enough soft tissue to cause tension-free closure and avoid implant extrusion [24]. This is where ADM is used; instead of lifting the local serratus fascia and rectus fascia defined by the lateral range of the pocket, ADM allows to actively shape the lower pole, including the ability to establish a critically important subcutaneous fold in an ideal location. ADMs are also used when there are viable but weakened or thin skin flaps after a mastectomy that are at risk of wound spread or necrosis, as well as when the subcutaneous and lateral nipple folds have been leveraged during the mastectomy. According to some authors, ADMs also have the added benefit of reducing the capsular contracture rate and improving surface roughness [25,26]. Postoperative pain and expansion rates remain without significant changes [27,28]. The first results of a recent prospective randomized controlled trial on breast reconstruction with ADM indicate a complication rate of 36%, of which about half are minor. Half of the complications require surgical intervention [29]. The analysis showed that inadequate integration of the ADM increased the incidence of infection. Poor integration has been noted and linked to an increase in breast size, expander size, initial tissue-expander fill volume, and obesity [29]. In addition to breast reconstruction, ADMs have also gained greater acceptance for breast revision surgery. Allergan (Allergan Inc., USA) presented a reoperation rate of 30% for patients after primary augmentation and a rate of 41% for patients after revision augmentation [30]. In some studies, ADM combined with oxidized, regenerated cellulose was also used as an aid to obtain better cosmetic results in partial breast reconstruction [31]. Gwak et al. reported the first improvemed aesthetic outcomes using human ADM as a filler in the BCS (breast-conserving surgery) of 117 breast cancer patients treated at the Division of Breast and Thyroid Surgical Oncology of St. Vincent’s Hospital (Seoul, Republic of Korea) [32]. Broyles et al. compare two commercial ADMs in breast reconstruction in a multicenter, prospective, randomized, control trial in 2021. The researchers presented results supporting the use of human acellular dermal matrices in implant-based breast reconstruction and demonstrated no significant difference in matrix-related complication rates between FlexHD Pliable (acellular hydrated dermis) and AlloDerm RTU (ready to use) [33]. There is still a field in research for aesthetic outcomes in surgical oncology with acellular dermal matrix. This biomaterial seems to be starting to become a standard in breast reconstructive surgery.

## 5. Hernia Repair

Abdominal surgery is often associated with major abdominal wall closure problems. Leaving aside anatomical differences, it is always a challenge in general surgery. Incisional hernias often occur after laparotomies [34]. Surgeons often operate in the abdominal cavity, where the fascia is weak to close the wound, combined with debris from the intestines. As an implant, mesh has always been vulnerable to infection. Mesh made of non-absorbable material is inapplicable in these cases, and temporary artificial mesh often does not provide optimal tensile strength to avoid the occurrence of hernias. Studies have shown that ADM is effective in reducing the rate of hernia recurrence in infected fields compared to primary closure without mesh [35,36]. The use of mesh materials in infected wounds results in a high incidence of mesh infection, fibrosis, fistulas, and adhesion formation. To avoid such complications, biomaterials obtained from animal or human sources are now being used [37]. Acellular dermal matrices are advertised as an alternative to man-made mesh material. The main aim of this application is to decrease the rates of infection, disintegration, extrusion, and rejection compared to artificial materials [37,38]. 

Lung hernia following minimally invasive cardiac surgery is rare, with few reported cases in the literature. Surgical repair is debated, and several methods have been described, including a variety of synthetic and biological materials. Stanizzi et al. presented successful reconstruction with six year follow-up on a patient with a lung hernia after a minithoracotomy. Stanizzi et al. suggest that ADMs are a safe and reliable surgical technique for lung hernia repair due to their biological and mechanical properties, even in those secondary hernias to minithoracotomy where complete muscle coverage of the matrix could not be provided [39]. Lightfoot et al.’s performed a retrospective cohort study comparing 40 consecutive patients who underwent open component separation (CS/VHR—Component Separation/Ventral Hernia Repair) with porcine ADM reinforcement to 39 consecutive patients who underwent open CS/VHR with bovine ADM [40]. Lightfoot et al. study showed that recurrences and complications requiring reoperation were fewer, which trended toward but did not reach statistical significance [40]. There is still room for further research in the field of ADM in hernioplasty. 

## 6. Reconstructive Gynecology

Human acellular dermal matrices (ADMs) can be used successfully in gynecological reconstructive surgery [41,42]. Ward et al. conducted a retrospective analysis of thirty-three women suffering from recurrent stage II anterior vaginal dislocations or primary or recurrent stage III-IV vaginal dislocations who underwent paravaginal vaginal reconstruction using commercially available AlloDerm between the years of 1998 -2002. The grafts of this commercial ADM product were placed over the base of the bladder and fixed with sutures to the fascia of the pelvic tendinous arch with four intermittent 2-0 sutures [43]. Among the 33 women who underwent the described reconstructive procedure, 20 were able to undergo regular annual clinical follow-up. Long-term data were obtained from 24 of the 33 patients (72.7%). The women did not seek urogynecologic care from other physicians. In conclusion, paraval-vaginal reconstructions with AlloDerm matrix have been shown to be safe and well-integrated by the recipient’s body [43].

Similar results were obtained by Clemons et al., where thirty-three women who underwent paravaginal vaginal reconstruction using the AlloDerm transplant were analyzed. Premature vaginal prolapse was assessed before surgery and every 6 months after surgery. Recurrence of prolapse, changes in functions (urinary incontinence, sexual activity), and complications have been observed. Clemens et al. showed that vaginal repair using the acellular dermal matrix in women with recurrent stage II or III/IV anterior vaginal wall prolapse is safe and has good results within the first two years of follow-up. [44]. Human acellular dermal matrix has also found application in laparoscopic surgery. Karon et al. performed a study on the effectiveness and satisfaction of patients after laparoscopic sacrocolpopexy using a non-crosslinked ADM (2019). Karon et al. showed that, among interviewed patients, 85.22% (n = 75/88) reported the treatment of their prolapse as “very successful” (57.95%; n = 51) or “moderately successful” (27.27%; n = 24). Another 9.09% (n = 8/88) reported the treatment as “somewhat successful", and only 5.68% (n = 5/88) reported the treatment was “not at all successful.” Similarly, 88.5% (n = 77) of interviewed patients reported their current health status as a “little better” (16.09%; n = 14) or “much better” (72.42%; n = 63), compared to how they were doing before the pelvic-floor operation. Another 6.9% (n = 6) reported they were “about the same”, and 4% (n = 4) reported that their health status was worse compared to their preoperative status [45]. Koron et al. conclude that the patient outcomes in their study, combined with results comparable to those reported with polypropylene mesh but without the erosion complications, make the ADM biologic patch a good alternative in laparoscopic sacrocolpopexy [45]. There is still a lack of further research in surgical urogynecology with ADM applications. Further research should be considered in this field.

## 7. Other Surgical Applications

**Figure 4:**
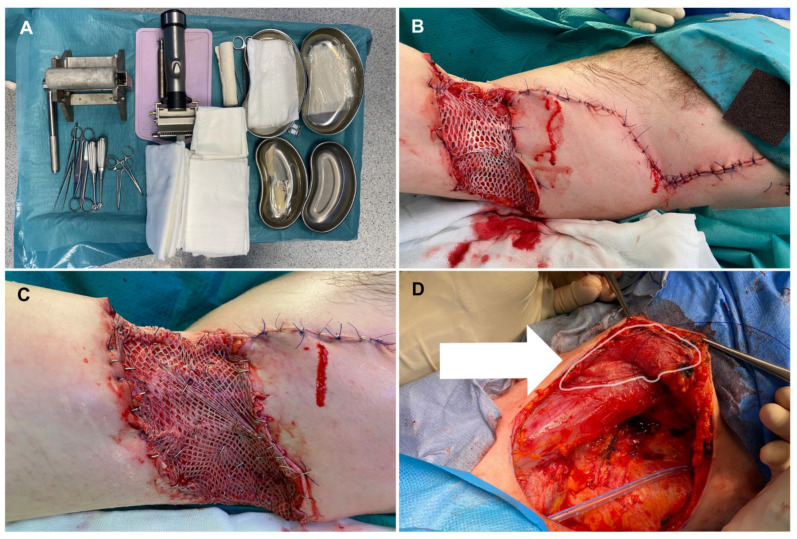
Multiple applications of ADM in Hidradenitis Suppurativa Surgery Reconstructions (own material). (**A**) Surgical equipment required for ADM and ADM-STSG co-graft surgery; photo shows surgical instruments—a meshing device, surgical tools, and the thawing process of our in-house ADM product; (**B**) ADM application to the wound (first step of co-graft); (**C**) Second step of co-graft—STSG fixation (STSG overlaps ADM); (**D**) ADM grafted to the skin flap—the ADM is pointed by an arrow and a contour (**B**,**C**) shows a 2-stage operation, which is a co-graft of ADM and STSG, (**D**) shows a different operation (ADM placement under the skin flap).

There are a few interesting novel applications of ADM. Lee Y et al. presented good results of the effect of ADM and STSG in the treatment of deep tissue defects at the donor site of free flaps [46]. They were co-grafting ADM and STSG in the donor site flaps with good aesthetic scar formation results. Chaffin et al. showed the use of ADM in the treatment of Hidradenitis Suppurativa. They implanted ADM in four cases followed by flap reconstruction, in two of the cases ADM was used as a skin substitute (biological dressing), and in 1 case ADM application was followed by skin grafts after 22 weeks of placement of the acellular dermal matrix [47]. However, the first co-graft of ADM and STSG after wide excisions of Hidradenitis suppurativa during one surgical procedure was performed by Gierek et al. (2022) [48]. ADM co-graft and other applications in hidradenitis suppurativa surgery are presented in Figure 4. A short summary of the literature review is in Table 2.

## 8. Conclusions

Since Livesey’s potentially useful concept of ADM [14], ADM has been used in a different new application like hidradenitis suppurativa surgery [47,48], ADM seems to be a biomaterial with great potential. The place of ADM in surgical specialties will extend, and we are sure that we will see a new surgical procedure with ADM. 

Acellular dermal matrices are providing alternatives to form an aesthetic and elastic scar. ADMs are taken into account in many surgical applications. There are new possible applications. ADMs found their place in reconstructive breast surgery and hernia repair surgery, which is the most studied area of ADM applications currently. However, there is still a great need for further research into new applications of ADM in reconstructive surgery. This manuscript should encourage the scientific community to further research and improve reconstruction methods with the use of ADM.

## Figures and Tables

**Table 1:** Examples of commercial ADM products available on the market. (ADM—acellular dermal matrix).

Name of the Product (Commercial ADM)	Manufacturer	Origin	General Characteristics
AlloDerm	Lifecell Corp., USA	Human dermis	Intended to be used for repair or replacement of damaged tissue
Cymetra	Lifecell Corp., USA	Human dermis	Micronized AlloDerm tissue for injections
NeoForm	Mentor, USA	Human dermis	Used in breast surgery
DermaMatrix	Musculoskeletal Transplant Foundation and Synthesis, USA	Human dermis	Rehydrates very quickly, does not require refrigerated storage
Glyaderm	Euroskinbank, NL	Human dermis	Depending on the dimension of the patient’s wound, donor skin is available from 40 cm^2^ to 150 cm^2^.
Permacol,	Tissue Science Laboratories, USA	Porcine dermis	Used in hernia repair. Available in large sheets for abdominal wall use, cross-linked for enhanced durability.
Matriderm	Skin and Healthcare, USA	Bovine dermis	Used for skin defects
Pelnac	Gunze Corp., JP	Bovine dermis	Used for skin defects
Renoskin	Perouse Plastie, FR	Bovine dermis	Silicone outer layer; used for skin defects

**Table 2:** Summary of the literature, brief chronological characteristics of manuscripts on acellular dermal matrix.

Year of Publication	Reconstruction	Author/Number of Reference	General Information
1995	Burns	Livesey et al. [14]	First concept of ADM
1995	Burns	Wainwright et al. [18]	First use of ADM in burns
2001	Breast surgery	Duncan et al. [25]	First use of ADM in breast surgery
2005	Breast reconstruction (surgical oncology)	Breuing et al. [23]	First use of ADM after mastectomies
2008	Hernia repair	Candage et al. [36]	ADM as an alternative in synthetic mesh in hernia repair
2014	Hernia repair	Garvey et al. [37]	ADM is preventing the infection comparing to the synthetic mesh
2003	Reconstructive gynecology	Clemons et al. [44]	Paravaginal reconstruction using the AlloDerm
2007 *	Reconstructive gynecology	Ward et al. [43]	Reconstruction of prolapsy with Alloderm
2022	Hidradenitis suppurativa	Gierek et al. [48]	First use of co-graft of ADM and STSG in hidradenitis suppurativa

* Data was collected in years 1998-2002, paper was published in 2007. ADM—Acellular Dermal Matrix, STSG—Split thickness skin graft.

## Data Availability

Not applicable.
